# Improved WOA-DBSCAN Online Clustering Algorithm for Radar Signal Data Streams

**DOI:** 10.3390/s25165184

**Published:** 2025-08-20

**Authors:** Haidong Wan, Cheng Lu, Yongpeng Cui

**Affiliations:** 1 School of Information Science and Engineering, Southeast University, Nanjing 211189, China; 230199239@seu.edu.cn; 2 School of Electronic Engineering, Xidian University, Xian 710071, China; w624531@163.com

**Keywords:** signal sorting, data flow, DBSCAN algorithm, improved whale optimization algorithm, online sorting

## Abstract

For the pulsed data streams emitted by multiple signal sources that generate aliasing, traditional density clustering algorithms have the problems of poor clustering effect, heavy reliance on manual experience to set the parameters, and the need to carry out density clustering every time new data are input, resulting in a huge amount of computation. Therefore, an online density clustering algorithm based on the improved golden sine whale optimization is proposed. First, by adding new parameters to the density clustering algorithm, the neighborhood is changed from a single parameter Eps to a joint decision of the parameters Eps and θ, which avoids cross-cluster expansion by more flexibly delimiting the neighborhood range. The improved golden sine whale optimization algorithm is then used to obtain the optimal parameter solution of the DBSCAN algorithm. Finally, the idea of flow clustering is introduced to determine whether a pulse belongs to a valid library, an outlier library, or an inactive library by comparing the distance between the input pulse and each cluster center, effectively reducing the number of pulses required for analysis. The experiment proves that the algorithm improves the sorting accuracy by 57.7% compared to the DBSCAN algorithm and 37.8% compared to the WOA-DBSCAN algorithm.

## 1. Introduction

Radiation source signal sorting is an important part of electronic reconnaissance, which is a prerequisite for electromagnetic target identification and localization. Through sorting, feature extraction, and de-interleaving, the processing of radiation source signals acquired by the receiver will distinguish the overlapping radiation source signals, thus realizing the purposes of radiation source identification and threat assessment, and obtaining the advantage of electromagnetic confrontation in modern war. As radar detection and signal processing technologies continue to evolve, radar waveform design is moving toward greater diversity and complexity, and radar signal countermeasure technologies are also becoming increasingly sophisticated. In this context, the radar signals received at the end show greater diversity [1] and non-stationarity in terms of pulse parameters, modulation methods, and spectral characteristics, which significantly increases the difficulty of feature separation in the signal sorting process. As a result, traditional clustering methods often exhibit issues such as blurred inter-class boundaries and intra-class feature fragmentation when processing such signals, leading to reduced accuracy in clustering results. This problem not only weakens the ability to determine the number of targets effectively but may also introduce biases in the identification and assessment of the opponent’s operational intentions, thereby adversely affecting the reliability of electronic countermeasures and battlefield situational awareness.

Traditional signal sorting is based on Pulse Repetition Interval (PRI) of the signal [2], but it becomes extremely difficult and inefficient when facing large-scale, multi-dimensional, and diverse data. As a result, the k-means clustering algorithm [3,4,5] came into the limelight. Rodriguez, M.Z., in paper [6], compares the k-means algorithm with other algorithms. However, the traditional k-means clustering algorithm requires the number of cluster centers to be set in advance and has limitations when dealing with non-convex-shaped clusters, noisy data, and unevenly distributed densities. To address this limitation, Martin Ester et al. proposed the density-based spatial clustering of applications with noise (DBSCAN) algorithm in [7] in 1996. References [8,9] extend the DBSCAN algorithm from the two directions of spectral adaptation and space-time density clustering, respectively. Reference [8] combines the spectral adaptation method with the traditional DBSCAN algorithm to dynamically adjust the pulse repetition period spectral pair threshold to accomplish the sorting of different types of PRI modulated signals. In contrast, Reference [9] proposes the ST-DBSCAN clustering algorithm based on the traditional DBSCAN algorithm for different signal arrival moments. The algorithm introduces a spatio-temporal neighborhood ΔT in DBSCAN, which effectively separates out the time-frequency domain overlapping pulse sequences, and is less affected by changes in the repetition intervals of the radiation source pulses. However, the algorithms proposed in [8] and [9] need to rely on the user’s personal experience to set the parameters.

Facing the two parameters of neighborhood radius Eps and minimum neighborhood points MinPts, which need to be set manually by the DBSCAN algorithm relying on experience, in recent years, more and more researchers have tried to combine machine learning with the DBSCAN clustering algorithm to obtain the optimal parameters [10,11,12,13]. Reference [14] used the whale optimization algorithm (WOA) combined with the DBSCAN algorithm to obtain the optimal parameters. However, the WOA algorithm is prone to falling into the local optimum, especially in the complex multi-peak function optimization, where it is difficult to find the global optimal solution, and with the increase in the problem dimension, the search space expands dramatically, and the performance of the algorithm decreases significantly.

Stream clustering is the process of real-time online “clustering” of radar source signals on a pulse-by-pulse basis when performing online sorting [15]. The STRAP (Stream Affinity Propagation) stream clustering algorithm was first proposed in [16] and applied to sensor networks to update the “clusters” with real-time evolution under data input. However, the AP clustering algorithm adopted by the STRAP algorithm in the pre-sorting stage needs to calculate the similarity between points, which is too computationally intensive in the face of large-scale datasets and is not good at dealing with noisy points. The DBSCAN algorithm is more efficient in the face of large-scale datasets and can effectively distinguish the noise points. Therefore, this paper adopts the DBSCAN algorithm combined with a stream clustering algorithm to sort data streams.

In this paper, we use the improved DBSCAN algorithm to sort the initial data in the signal level sorting stage, and the improved golden sine whale optimization algorithm (IGWOA) is used to obtain the optimal solution of the parameters in the DBSCAN algorithm and to calculate the cluster centers of each cluster. After the first level of sorting is completed, the distance between the new input data and each cluster center is calculated and sorted, and the cluster centers and database are updated. In Section 3, this sorting algorithm is compared with the WOA-DBSCAN algorithm and the DBSCAN algorithm to verify the effectiveness of the algorithm. In Section 4, the full paper is summarized.

## 2. Improvement of WOA-DBSCAN Online Clustering Algorithm

**Figure 1:**
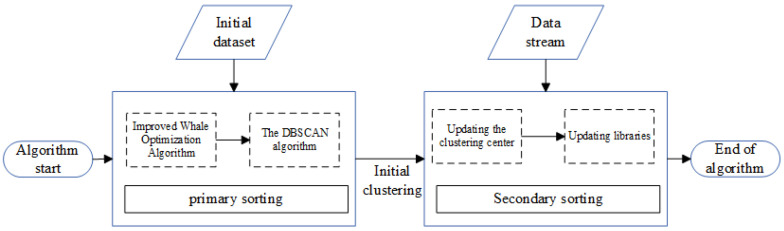
Algorithm framework diagram.

In the face of multiple signal sources, transmitting generates an aliased data stream. The IGWOA-DBSCAN clustering algorithm is first used to perform first-level binning of the initial data and to calculate the clustering centers. During the first-level sorting, the IGWOA optimization algorithm is used to estimate the DBSCAN parameters, since the DBSCAN algorithm relies on human experience for parameter setting. After completion of the first-level sorting, to enter the second-level sorting stage, each pulse of the input is compared with each clustering center, and online sorting is performed by the distance from each clustering center. The algorithm is framed in Figure 1.

### 2.1. Parameter Selection Based on the Improved Whale Optimization Algorithm

The improved DBSCAN clustering algorithm introduces a new parameter θ, compared to the traditional DBSCAN algorithm, and therefore relies more on manual experience for parameter setting, and the uncertainty of the sorting results increases. In this section, the improved whale optimization algorithm [17,18,19] is used to obtain the parameter settings of the DBSCAN algorithm to avoid the intervention of manual experience.

When faced with an unknown parameter, it is first necessary to select a parameter range. The planar distance between each point and the other M-1 points is found, and an M×M matrix is constructed for M data points. Find the median for each column of the matrix and obtain a vector of 1×M. The median of this vector is chosen as the upper bound of $Eps_{ub}$ and the lower bound $Eps_{lb}$ is the minimum value of this vector. The selection methodology for both the upper and lower bounds of the range θ is consistent. After selecting the parameter ranges of Eps and θ, the maximum and minimum values of the two parameters are selected to form the neighborhood $\varepsilon_{ub}$ and $\varepsilon_{lb}$ respectively. Calculate the number of data points in the neighborhood $\varepsilon_{ub}$ and neighborhood $\varepsilon_{lb}$ for each data point and find the mean values $E_{ub}$ and $E_{lb}$, which are used as the upper and lower bounds $MinPts_{ub}$ and $MinPts_{lb}$, respectively, for parameter MinPts.

Initiate the positioning of the initial whale stock following the delineation of the range.

(1) $$x_{str}\left(1\right)=rand()\times \left(eps_{ub}-eps_{lb}\right)+eps_{lb}$$

(2) $$x_{str}\left(2\right)=rand()\times \left(MinPts_{ub}-MinPts_{lb}\right)+Minpts_{lb}$$

(3) $$x_{str}\left(3\right)=rand()\times \left(\theta_{ub}-\theta_{lb}\right)+\theta_{lb}$$

(4) $${\vec{X}}_{str}^{j}={\left[x_{str}\left(1\right),x_{str}\left(2\right),x_{str}\left(3\right)\right]}^{T}$$

Equation (4) is where ${\vec{X}}_{str}^{j}$ is the initial position of the j-th whale population, j=1,2,…Ps, and Ps is the number of whale populations. Subsequently, establish the upper iteration limit T. An increased ratio of the upper iteration limit T to the population size PS enhances the probability of attaining a globally optimal solution. The WOA algorithm treats the current optimal solution as the target prey, and after learning the location of the prey, the whales begin to surround the prey by starting to update their position based on the current target position.

(5) $$\vec{X}\left(t+1\right)={\vec{X}}^{*}\left(t\right)-\vec{A}\cdot \vec{D}$$

(6) $$\vec{D}=\left|\vec{C}\cdot {\vec{X}}^{*}\left(t\right)-\vec{X}\left(t\right)\right|$$

Equation (5) is the position update formula, ${\vec{X}}^{*}\left(t\right)$ is the prey position at the current moment t, and $\vec{D}$ is the distance between the prey position and the current whale group. $\vec{A}$ in Equation (5) and $\vec{C}$ in Equation (6) are coefficient vectors.

(7) $$\vec{A}=2{\vec{a}}_{1}\cdot {\vec{r}}_{1}-{\vec{a}}_{1}$$

(8) $$\vec{C}=2\cdot {\vec{r}}_{2}$$

In Equations (7) and (8), ${\vec{r}}_{1}$ and ${\vec{r}}_{2}$ denote two random numbers between 0 and 1. ${\vec{a}}_{1}$ in Equation (7) is a nonlinear decrease from 2 to 0 parameter factor with iterations.

(9) $$\vec{a}=2-\frac{2}{e^{-0.1\left(t-T\right)}}$$

**Figure 2:**
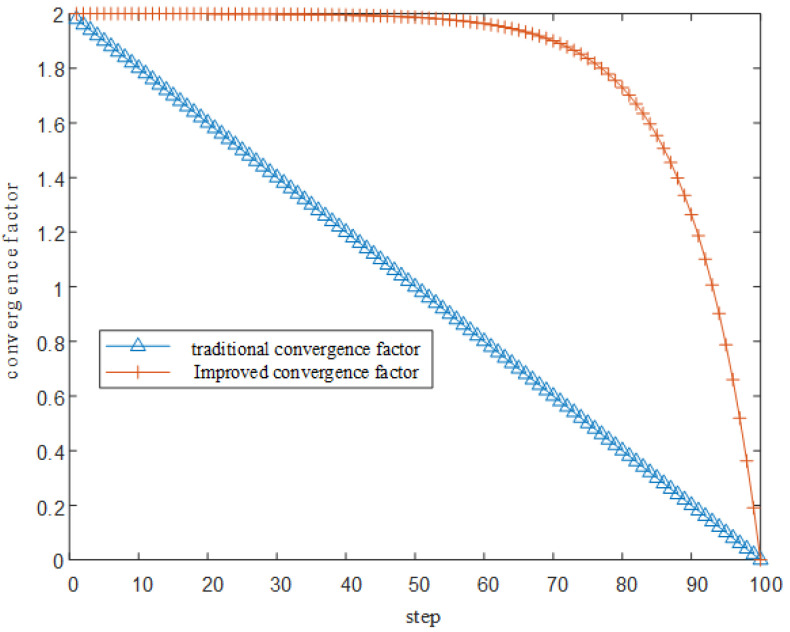
Comparison curve of the traditional convergence factor and the improved convergence factor.

Plotting the nonlinear convergence factor curve against the conventional convergence factor curve is shown in Figure 2.

The above figure shows the traditional convergence factor curve compared to the improved convergence factor curve. From the above figure it can be seen that the improved convergence factor compared to the traditional convergence factor keeps the value of $\vec{a}$ at a high level in the early and middle stages of the iterative process to ensure the global search capability of the algorithm, and decreases rapidly in the later stages of the iteration to ensure that the algorithm concentrates on a local search.

**Figure 3:**
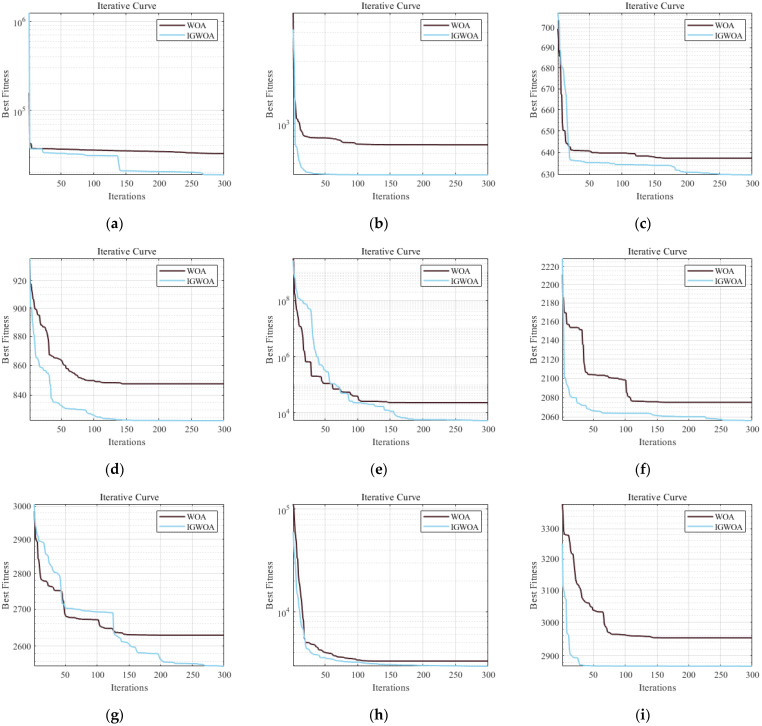
Comparison of the traditional convergence factor and the improved convergence factor test in 10-dimensional conditions. (**a**) Test function F1 comparison curve; (**b**) Test function F2 comparison curve; (**c**) Test function F3 comparison curve; (**d**) Test function F4 comparison curve; (**e**) Test function F6 comparison curve; (**f**) Test function F7 comparison curve; (**g**) Test function F9 comparison curve; (**h**) Test function F11 comparison curve; (**i**) Test function F12 comparison curve.

The WOA optimization algorithm using the traditional convergence factor and the improved convergence factor is tested using the CEC2022 function test set in 10 dimensions, and the test results are shown in Figure 3.

Equation (9) is where t is the number of current iterations and T is the total number of iterations. To avoid falling into a local optimum too early, a stochastic search method is used when $\left|A\right|>1$.

(10) $$\vec{X}\left(t+1\right)={\vec{X}}_{rand}\left(t\right)-\vec{A}\cdot \vec{D}$$

(11) $$\vec{D}=\left|\vec{C}\cdot {\vec{X}}_{rand}\left(t\right)-\vec{X}\left(t\right)\right|$$

It can be seen that the stochastic search method is highly similar to the formulae used when encircling prey, with the only difference being that the location of the current prey is changed from the location of the optimal solution, ${\vec{X}}^{*}\left(t\right)$, to a random whale, ${\vec{X}}_{rand}\left(t\right)$.

To simulate the process of a humpback whale’s foaming net attack, a spiral equation is created between the target and the whale.

(12) $$\vec{X}\left(t+1\right)={\vec{X}}^{*}\left(t\right)\cdot \left|\sin\left({\vec{r}}_{3}\right)\right|+{\vec{r}}_{4}\cdot \sin\left(r_{3}\right)\cdot \left|q_{1}\cdot \vec{X}\left(t\right)-q_{2}\cdot {\vec{X}}^{*}\left(t\right)\right|$$

(13) $$q_{1}=-\pi +\left(1-\Gamma \right)\times 2\pi$$

(14) $$q_{2}=-\pi +\Gamma \times 2\pi$$

Equation (12), is where ${\vec{r}}_{3}$ is a uniformly distributed random point between $\left[0,2\pi \right]$ that determines the distance to move in the next iteration, and ${\vec{r}}_{4}$ is a random point uniformly distributed in the range $\left[0,\pi \right]$ that determines the direction of the position update of the individual in the next iteration. Both $q_{1}$ and $q_{2}$ are coefficients obtained by introducing the golden section number [20]; these coefficients reduce the search space so that the individual converges to the optimal solution, the golden section coefficient $\Gamma =\left(\sqrt{5}-1\right)/2$.

Encircling prey and attacking prey are chosen by random means, so the mathematical expression is given by:

(15) $$\left\{\begin{array}{l} \vec{X}\left(t+1\right)={\vec{X}}^{*}\left(t\right)-\vec{A}\cdot \vec{D},p<0.5\\ \vec{X}\left(t+1\right)={\vec{X}}^{*}\left(t\right)\cdot \left|\sin\left({\vec{r}}_{3}\right)\right|+{\vec{r}}_{4}\cdot \sin\left(r_{3}\right)\cdot \left|q_{1}\cdot \vec{X}\left(t\right)-q_{2}\cdot {\vec{X}}^{*}\left(t\right)\right|,p\geq 0.5 \end{array}\right.$$

Equation (15), is where p is a random number between (0,1). To balance the global and local searches, the adaptive linear inertia weights w in the particle swarm algorithm are improved to be nonlinear, which is used to achieve the balance between global and local searches [21]. Introducing this into Equation (16), it is given as:

(16) $$\left\{\begin{array}{l} \vec{X}\left(t+1\right)=w{\vec{X}}^{*}\left(t\right)-\vec{A}\cdot \vec{D},p<0.5\\ \vec{X}\left(t+1\right)=w{\vec{X}}^{*}\left(t\right)\cdot \left|\sin\left({\vec{r}}_{3}\right)\right|+{\vec{r}}_{4}\cdot \sin\left(r_{3}\right)\cdot \left|q_{1}\cdot \vec{X}\left(t\right)-q_{2}\cdot {\vec{X}}^{*}\left(t\right)\right|,p\geq 0.5 \end{array}\right.$$

(17) $$w=\sin(\frac{\pi t}{2T}+\pi )+1$$

**Figure 4:**
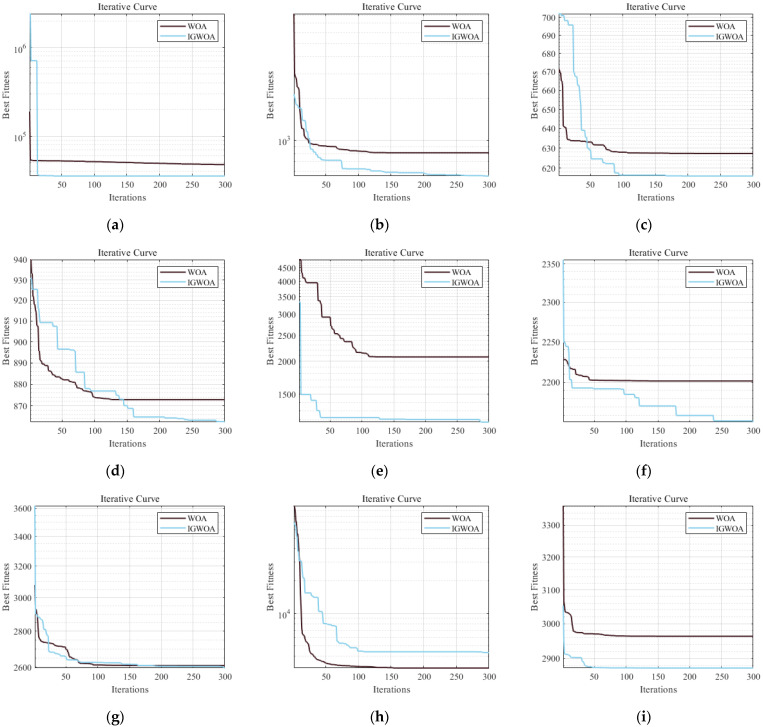
Comparison of the improved golden sine whale optimization algorithm and the traditional whale optimization algorithm tests under 10-dimensional conditions. (**a**) Test function F1 comparison curve; (**b**) Test function F2 comparison curve; (**c**) Test function F3 comparison curve; (**d**) Test function F4 comparison curve; (**e**) Test function F6 comparison curve; (**f**) Test function F7 comparison curve; (**g**) Test function F9 comparison curve; (**h**) Test function F11 comparison curve; (**i**) Test function F12 comparison curve.

The improved golden sine optimized whale optimization algorithm is tested against the traditional whale optimization algorithm using the CEC2022 function test set, and the test results are shown in Figure 4.

After the whale’s current position is updated, it is then compared to the range of whale positions obtained, and if the current whale position is greater than the upper range boundary or less than the lower range boundary, the current position is changed to the upper range boundary or lower range boundary to avoid crossing the boundary. Post-update of the whale’s position, the current coordinates are integrated into the enhanced DBSCAN algorithm, utilizing contour coefficients to ascertain the fitness of the present location. The fitness formulation employed in Reference [13] is given by:

(18) $$S\left(i\right)=\frac{b\left(i\right)-a\left(i\right)}{\max\left\{a\left(i\right),b\left(i\right)\right\}}$$

In Equation (18), $a\left(i\right)$ is the average distance from the i-th data point to the other data points in the cluster class to which it belongs, and $b\left(i\right)$ is the average distance from the i-th data point to the data points in all other clusters except the cluster to which i belongs. The closer $S\left(i\right)\in \left[-1,1\right]$ and $S\left(i\right)$ are to 1, the higher the quality of the clustering. The algorithm is changed to count the number of correct points of each radar sorting, and the number of correct points of all radars is summed and divided by the total number of data points to obtain the adaptability parameter, which ranges from $\left[0,1\right]$; the closer to 1, the better the sorting effect. Should the fitness of the current whale’s position surpass that of the present optimal solution, the position of the latter is revised to align with that of the former. The Algorithm 1 is shown as follows:
**Algorithm 1:** Improved whale optimization algorithmInput: the whale population $x_{i}$ (i = 1, 2,…,n), Number of iterations T;   Fitness calculation rules S();1: z = find(max(F($x_{i}$)));2: $X^{*}$=$X_{Z}$;3: t = 1;4: **While**(t < T)5: **for** each search agent;6: Generate A, C, and a based on position, randomly generate parameters 1 and p;7: **if1**(*p* < 0.5);8:  **if2**(|A| < 1);9:   $X_{k}^{j+1}=wX_{k}^{*}-A\cdot D_{k}$; 10:  **else**;11:   $X_{k}^{j+1}=X_{noul}\left(t\right)-A\cdot D_{k}$
12:  **end if2;**13: **else;**14:   $X_{K}^{j+1}=w{\vec{X}}^{*}\left(t\right)\cdot \left|\sin\left({\vec{r}}_{3}\right)\right|+{\vec{r}}_{4}\cdot \sin\left(r_{3}\right)\cdot \left|q_{1}\cdot \vec{X}\left(t\right)-q_{2}\cdot {\vec{X}}^{*}\left(t\right)\right|$
15: **end if1**16: **end for**17: Update when optimal solution $X^{*}$;18: t = t + 1;19: **end while**Output $X^{*}$


### 2.2. Signal-Level Binning Based on the IGWOA-DBSCAN Clustering Algorithm

The traditional DBSCAN clustering algorithm relies heavily on manual experience to set parameters. Therefore, this section proposes the IGWOA-DBSCAN algorithm based on the traditional DBSCAN algorithm combined with the golden sine whale optimization algorithm above, which introduces a new variable in addition to the parameters Eps and MinPts of the traditional DBSCAN clustering algorithm to flexibly adjust the range of the neighborhood, and obtains the optimal values of the parameters by using the IGWOA algorithm.

The DBSCAN clustering algorithm is a classical algorithm based on density clustering [22,23,24], which identifies clusters of arbitrary shape and rejects noise by defining local density features. The algorithm first initializes two parameters, Eps and MinPts, which are used to define the neighborhood range and determine the core object, respectively. In the algorithm flow, all data points are initially marked as unvisited and then traversed one by one. For the current point, if its Eps neighborhood contains at least MinPts points, it is marked as the core point and recursively extends the cluster based on it, by collecting density-accessible neighboring points, and gradually incorporating directly density-accessible and density-connected points into the same cluster; on the contrary, if there are not enough neighboring points, it is temporarily marked as noise. In this process, the core points form the main structure of the cluster through neighborhood expansion, the boundary points are attached to the core points and are grouped into clusters, and the noise points are excluded because they cannot satisfy the density condition.

The traditional DBSCAN algorithm performs well on data with a general geometric regular distribution, but the effect of spatial density clustering is doubtful when the data distribution between dimensions is significantly discrete, or when some data have special significance.

**Figure 5:**
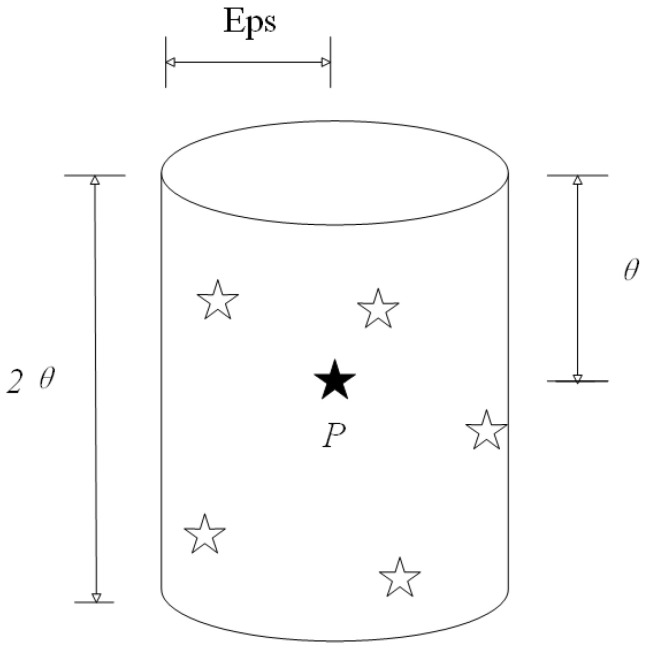
Spatial neighborhoods of spatial entities.

For the same radar signals emitted by multiple identical units, their carrier frequency, bandwidth, and other parameters are similar. Therefore, in this paper, for the above situation, based on the pulse aliasing signals emitted by multiple identical units, three of the PDW parameters are selected, and new parameters are introduced based on the neighborhood determination of the traditional DBSCAN algorithm. The neighborhood is shown in Figure 5.

**Figure 6:**
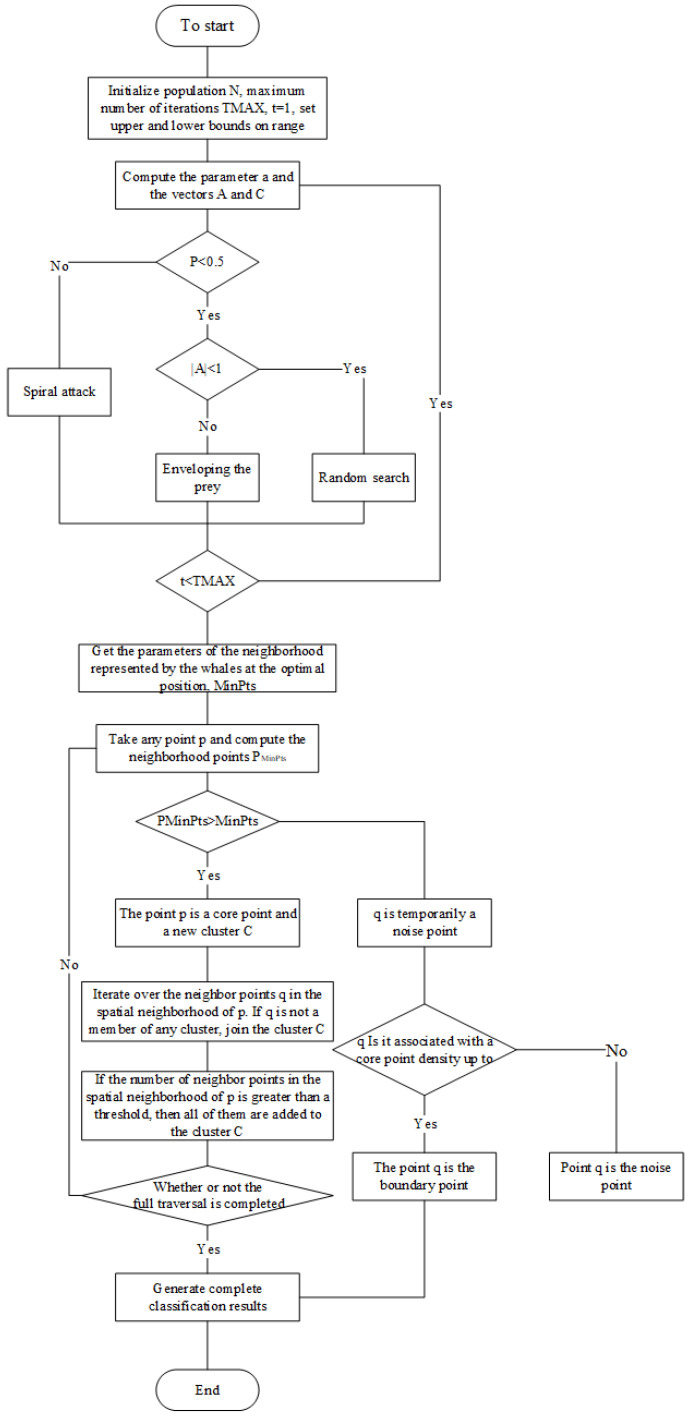
Flowchart of the IGWOA-DBSCAN algorithm.

The black star in Figure 5 represents the current point, and the white stars represent data points within the neighborhood of the current point. If the number of white stars is greater than or equal to the threshold, the black star is the core point. The spatial neighborhood of this method is jointly determined by two parameters, θ and Eps, which enables more flexible delineation of the neighborhood range. The method is able to adjust the range of spatial neighborhoods more flexibly by excluding some points that would otherwise be included in traditional neighborhoods, avoiding the problem of cross-cluster development that may occur during sorting. Different from the traditional spatial density clustering idea, the spatial ε-neighborhood and MinPts parameters are calculated using the IGWOA optimization algorithm. This is performed by iterating each individual in the cluster until the upper iteration point is reached or the optimal point is obtained, and the parameters represented by the optimal whale are recorded, i.e., the optimal spatial neighborhood and the optimal MinPts parameters. After obtaining the DBSCAN parameters, traversing to check each point, starting from any core point, and continuously expanding to the density reachable spatio-temporal neighborhood ε, a maximized region containing the core and boundary points is obtained, which is regarded as a clustered cluster, and any two points within the cluster are at least density-connected. After the statistics for all the core points are completed, the objects that are still not clustered into any of the clusters are considered as noise points and are added to the noise bank for comparison with the subsequent input signals. The flowchart of the algorithm is shown in Figure 6.

### 2.3. Secondary Sorting Algorithm for Data Stream Signals

The DBSCAN clustering algorithm has the advantage that it does not need to specify the number of clustering centers for the pulsed data streams emitted by multiple sources. However, when dealing with streaming signals, the clustering calculation needs to be performed every time new data are input, and the amount of computation is huge. Therefore, the stream clustering algorithm [25,26,27] is used to deal with data stream signals.

In this section, we will introduce how to perform online sorting of the signals, which belongs to the second-level sorting algorithm in the whole sorting algorithm. Before the second-level sorting, we need to sort the initial data set through the first-level sorting composed of Section 2.1 and Section 2.2, to obtain the number of sorting clusters and the clustering center of each sorting cluster, which provides support for the second-level sorting in this section, and helps to judge which sorting cluster in which library the input signal should be sorted into. The flow clustering algorithm is divided into two steps. Step 1 is to preprocess the initial dataset using the IGWOA-DBSCAN clustering algorithm and calculate the corresponding clustering centers for each cluster obtained. The clustering center is obtained by summing the pulses in each of the sorting clusters obtained during pre-sorting, respectively, and dividing by the number of pulses in that sorting cluster to obtain the clustering center of that sorting cluster. Step 2 performs pulse-by-pulse online binning matching on the pulsed radar signals. If the pulse signal cannot be matched with the clusters in the valid and inactivated pools, it is treated as an anomalous outlier. When the number of anomalous outliers exceeds a certain threshold, it is necessary to re-call the static clustering and update the summary library. In this paper, we introduce the concepts of “cluster validity”, “cluster inactivation,” and “cluster resurrection”. The entire cluster sorting process categorizes clusters into three evolutionary forms: effective, inactivated, and revived [28]. The clusters have an effective library $B_{e}$, an inactive library $B_{i}$, and an outlier library $B_{o}$.

When analyzing the current signal, only the effective library can be analyzed. When analyzing past signals, only the inactive library can be analyzed. When analyzing noise, only the outlier library can be analyzed. The creation of effective, inactive, and outlier libraries greatly reduces the number of pulses to be analyzed.

Let there be K clusters in the electromagnetic environment, with an effective library of binned clusters $\delta_{i-1}^{t},\delta_{i}^{t}$ at time t, an effective library of binned clusters $\delta_{1}^{t},\delta_{2}^{t},\ldots \delta_{i-2}^{t},\delta_{i+1}^{t},\delta_{k}^{t}$, and an outlier library of N signal pulses $P_{o}^{1},P_{o}^{2},\ldots P_{o}^{N}$.

When pulse $P^{t}$ flows in, the distance between the pulse and the center of mass of each sorting cluster is compared, and if the distance to more than one center of mass is less than a threshold value, the sorting cluster belonging to the closest center of mass is joined, and the sorting clusters and centers are updated. If the sorting cluster joined by this pulse belongs to an effective library, then Equation (19) applies:

(19) $$\left\{\begin{array}{l} \delta_{i-1}^{t},\delta_{i}^{t}\in B_{e}\\ \delta_{1}^{t},\delta_{2}^{t},\ldots ,\delta_{i-2}^{t},\delta_{i+1}^{t},\ldots ,\delta_{k}^{t}\in B_{i}\\ P_{o}^{1},P_{o}^{2},\ldots ,P_{o}^{N}\in B_{o} \end{array}\right.$$

If the sorting cluster joined by this pulse belongs to the inactivation library, update the sorting cluster with the center of mass and add the sorting cluster to the effective library. Assuming that the joined sorting cluster is $\delta_{1}^{t}$, then Equation (20) applies:

(20) $$\left\{\begin{array}{l} \delta_{1}^{t},\delta_{i-1}^{t},\delta_{i}^{t}\in B_{e}\\ \delta_{2}^{t},\ldots ,\delta_{i-2}^{t},\delta_{i+1}^{t},\ldots ,\delta_{k}^{t}\in B_{i}\\ P_{o}^{1},P_{o}^{2},\ldots ,P_{o}^{N}\in B_{o} \end{array}\right.$$

If the distance between this pulse and each of the sorting clusters is greater than the threshold, the pulse is added to the outlier library. If the number of pulses in the outlier library at this point does not exceed the number threshold, then Equation (21) applies:

(21) $$\left\{\begin{array}{l} \delta_{i-1}^{t},\delta_{i}^{t}\in B_{e}\\ \delta_{1}^{t},\delta_{2}^{t},\ldots ,\delta_{i-2}^{t},\delta_{i+1}^{t},\ldots ,\delta_{k}^{t}\in B_{i}\\ P_{o}^{1},P_{o}^{2},\ldots ,P_{o}^{N},P_{o}^{N+1}\in B_{o} \end{array}\right.$$

If the number of outlier library pulses exceeds a quantity threshold after the addition of this pulse, the outlier library is sorted, and the successfully sorted pulses are moved out of the outlier library, and the newly sorted clusters with successful sorting are added to the effective library.

(22) $$\left\{\begin{array}{l} \delta_{i-1}^{t},\delta_{i}^{t},\delta_{k+1}^{t}\in B_{e}\\ \delta_{1}^{t},\delta_{2}^{t},\ldots ,\delta_{i-2}^{t},\delta_{i+1}^{t},\ldots ,\delta_{k}^{t}\in B_{i}\\ \emptyset =B_{o} \end{array}\right.$$

**Figure 7:**
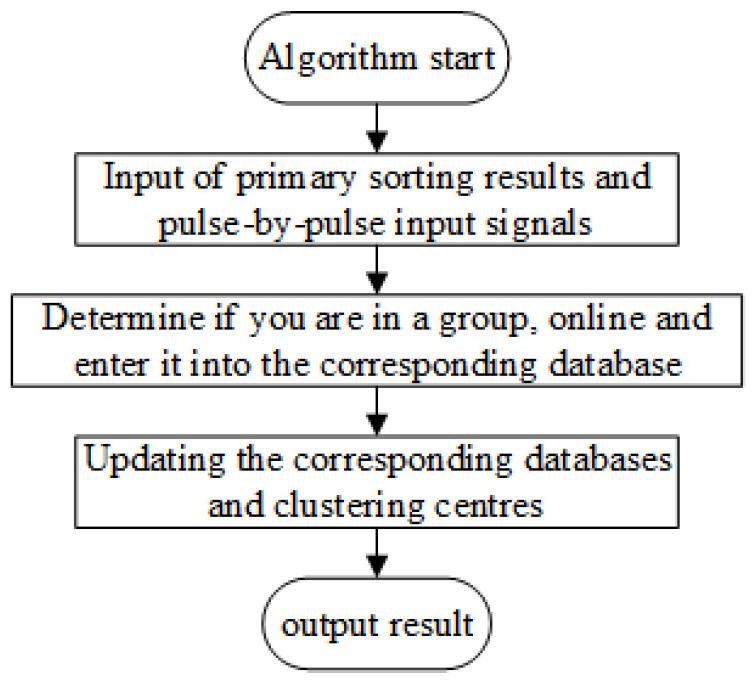
Secondary sorting method flow.

The secondary sorting algorithm is shown in Figure 7.

The stream cluster sorting steps are given as follows:

Step 1: Select the data of the previous $T_{0}$ time point to pre-sort and calculate the clustering center using the improved IGWOA-DBSCAN-based clustering algorithm as the initial information of the effective library. The center of clustering is calculated by summing the pulses in each of the sorting clusters obtained during pre-sorting separately and dividing by the number of pulses in that sorting cluster to obtain the center of clustering for that sorting cluster. For example, the sorting cluster 1 contains N pulses, and pulse $x_{i}$ contains $\left[\begin{array}{cccc} x_{i1} & x_{i2} & \ldots & x_{ij} \end{array}\right]$ total of j features, where i = 1…N. The formula for calculating the clustering center $xj_{1}$ is as follows:

(23) $$xj_{1}=\frac{1}{N}\sum_{i=1}^{N}x_{i}$$

Step 2: After the initialization is complete, each incoming pulse is sorted. Determine whether a point is in or out of a cluster by the distance between the current point and all cluster centers. Determine if the point is an in-cluster point by whether the distance between the point and all the cluster centers in the effective and inactivated libraries exceeds a threshold value. If it is less than the threshold, it is an in-cluster point, and the closest cluster center is selected and added to the cluster to which the cluster center belongs, and the cluster center of the cluster is updated. If the cluster belongs to an effective library, the effective library information is updated. If the cluster belongs to the inactivated library, update the cluster center and add the cluster to the effective library at the same time. If the distance between the point and all other clustering centers is greater than the threshold, then it is an outlier, and the outlier library is updated.

Step 3: Unknown clustering cluster emergence detection is performed on the outlier library, and if the number of samples in the outlier library exceeds a threshold or meets other triggering conditions, sorting is performed. If new clusters can be formed, the sorting results are entered into the effective library for updating.

Step 4: Inactivation detection is performed on the summary of clusters in the effective library. If no new samples are added to the cluster for more than a certain period, the cluster is inactivated and is moved from the effective library to the inactivated library for evolutionary updating.

Step 5: Resurrection detection is performed on the summary of cluster clusters in the inactivation library; if a certain number of new sample points are added to the inactivated cluster clusters within a certain period, resurrection of the cluster cluster occurs and the cluster cluster is moved from the inactivated library to the effective library, and the summary is updated in terms of evolution.

## 3. Results

In this section, the IGWOA optimization algorithm from Section 2.1 is used to obtain the DBSCAN algorithm proposed in Section 2.2 during simulation. When the population size of the optimization algorithm is small, although it has the advantages of a light computational burden and high efficiency in each iteration, its limited exploration range may lead to insufficient exploration and easily result in getting stuck in a global suboptimal solution. To compensate for the limited exploration capability of small populations, it is typically necessary to significantly increase the number of iterations. When the population size is large, although it can search a larger space in a single iteration, it has stronger global exploration potential and reduces the risk of prematurely getting stuck in a local optimum, but it significantly increases the time required for a single iteration. Therefore, when setting the number of iterations and population size for an optimization algorithm, it is important to avoid the high computational burden of a single iteration caused by an excessively large population size, as well as the inability to obtain a global optimal solution due to an insufficient number of iterations.

### 3.1. Verification of the Effectiveness of the First-Level Sorting Algorithm

To verify the feasibility of the first-level sorting algorithm. The simulation is performed using the data used in [29] consisting of 5 radar PDWs with a total of 1000 pulses, as shown in Table 1, and the data submitted in Science Data Bank [30] with a total of 1015 pulses consisting of 8 radar PDWs, after morphing as shown in Table 2, respectively, and both datasets contain the pulse width, the carrier frequency, and the angle of arrival.

Using the above dataset, the first-level binning is performed and compared with the DBSCAN as well as the WOA-DBSCAN algorithms. The DBSCAN algorithm with the WOA-DBSCAN algorithm uses the DBSCAN algorithm that contains only two parameters, Eps and MinPts, while the IGWOA-DBSCAN algorithm uses the DBSCAN algorithm proposed in this paper that contains three parameters, Eps, MinPts, and Δθ. When sorting all five radars in Dataset 1, the WOA and IGWOA optimization algorithms were used to find the optimal parameters for the DBSCAN algorithm proposed in Section 2.2. Both optimization algorithms were set to 100 iterations with a population size of 20. The parameters obtained using the WOA optimization algorithm were (23.5335, 18, 5), while those obtained using the IGWOA optimization algorithm were (14.5608, 91, 4). The standard DBSCAN parameters were set to (18, 80). The parameters obtained from WOA and IGWOA were input into the DBSCAN algorithm proposed in Section 2.2, while the standard DBSCAN parameters were input into the standard DBSCAN algorithm. The simulation results are as follows.

**Figure 8:**
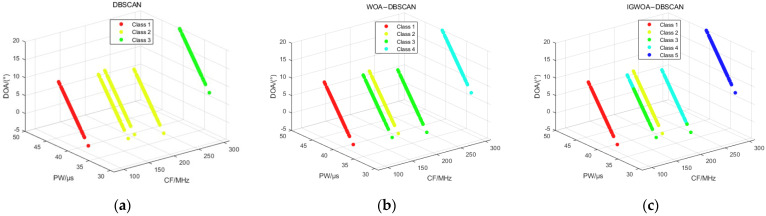
Dataset 1 sorting results. (**a**) DBSCAN algorithm sorting results; (**b**) WOA-DBSCAN algorithm sorting results; (**c**) IGWOA-DBSCAN algorithm sorting results.

Figure 8 shows the binning results of the DBSCAN, WOA-DBSCAN, and IGWOA-DBSCAN algorithms for dataset 1. Subfigure a shows the sorting results of the DBSCAN algorithm, which sorted out three radars in the dataset consisting of pulses from five radars. Subfigure b, on the other hand, shows the binning result of WOA-DBSCAN, which binned four radars in the dataset. And subfigure c shows the sorting results of the IGWOA-DBSCAN algorithm, which show that the algorithm sorts out five radars in the dataset, but there is a sorting error for some of the pulses of radar 3 and radar 4.

The statistics of the sorting accuracy when the three algorithms DBSCAN, WOA-DBSCAN, and IGWOA-DBSCAN are used for dataset 1, and are shown in Table 3.

**Figure 9:**
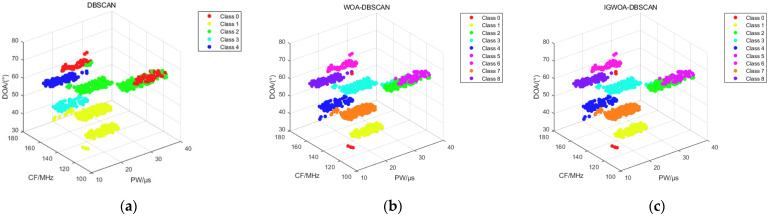
Sorting results of 3% interference pulses for 8 radars in dataset 2. (**a**) DBSCAN algorithm sorting results; (**b**) WOA-DBSCAN algorithm sorting results; (**c**) IGWOA-DBSCAN algorithm sorting results.

**Figure 10:**
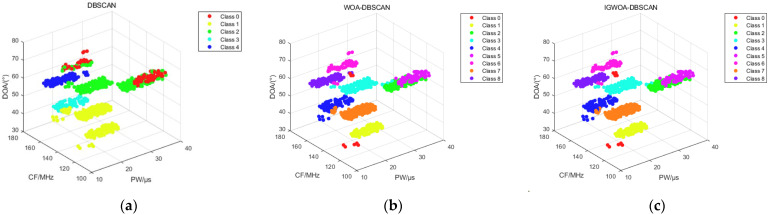
Sorting results of 5% interference pulses for 8 radars in dataset 2. (**a**) DBSCAN algorithm sorting results; (**b**) WOA-DBSCAN algorithm sorting results; (**c**) IGWOA-DBSCAN algorithm sorting results.

**Figure 11:**
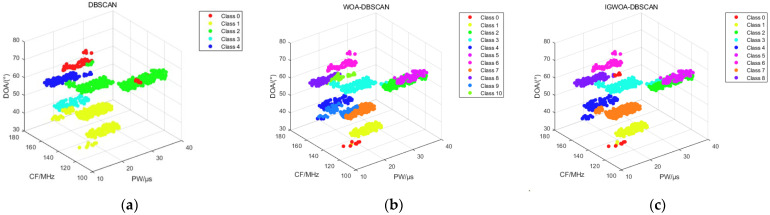
Sorting results of 10% interference pulses for 8 radars in dataset 2. (**a**) DBSCAN algorithm sorting results; (**b**) WOA-DBSCAN algorithm sorting results; (**c**) IGWOA-DBSCAN algorithm sorting results.

The 3%, 5%, and 10% interference pulses are added to dataset 2 and simulated using the DBSCAN, WOA-DBSCAN, and IGWOA-DBSCAN algorithms, respectively. The 3%, 5%, and 10% interference pulses are generated at the lower bound of each radar parameter, as given in Table 2, with a corresponding number of interference pulses obeying a uniform random distribution at the lower bound of the parameter. When sorting dataset 2 under different interference pulses, the WOA and IGWOA optimization algorithms are used to find the optimal parameters for the DBSCAN algorithm proposed in Section 2.2. Both optimization algorithms were set to 100 iterations with a population size of 20. Under 3% interference pulse, the parameters obtained using the WOA optimization algorithm were (7.6745, 20, 5), while those obtained using the IGWOA optimization algorithm were (7.2959, 28, 5). The standard DBSCAN parameters were set to (10, 80). Under 5% interference pulse, the parameters obtained using the WOA optimization algorithm were (7.6911, 24, 5), while those obtained using the IGWOA optimization algorithm were (7.5508, 34, 5). The standard DBSCAN parameters were set to (10, 80). Under 10% interference pulses, the parameters obtained using the WOA optimization algorithm are (7.2033, 28, 4), while those obtained using the IGWOA optimization algorithm are (8.0251, 30, 3). The standard DBSCAN parameters are set to (10, 80). The parameters obtained by WOA and IGWOA were respectively input into the DBSCAN algorithm proposed in Section 2.2, and the standard DBSCAN parameters were input into the standard DBSCAN algorithm, yielding Figure 9, Figure 10 and Figure 11, respectively.

Figure 9, Figure 10 and Figure 11 show the sorting results of the DBSCAN, WOA-DBSCAN, and IGWOA-DBSCAN algorithms when 3%, 5%, and 10% interfering pulses are added to dataset 2, respectively. The sorting accuracy of the DBSCAN, WOA-DBSCAN, and IGWOA-DBSCAN sorting algorithms with 3%, 5%, and 10% disturbing pulses, respectively, added to dataset 2, is counted and simulated, and the simulation results are shown below.

**Figure 12:**
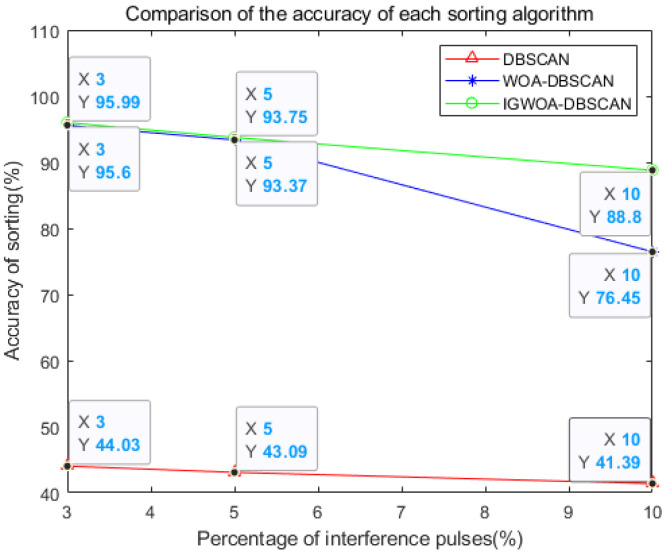
Comparison of the accuracy of each sorting algorithm.

From Figure 12, it can be seen that the sorting result of the IGWOA-DBSCAN algorithm is better than that of the WOA-DBSCAN algorithm when the same number of interfering pulses is added, while the sorting result of the WOA-DBSCAN algorithm is better than that of the DBSCAN algorithm. The sorting results of the same algorithm with different numbers of interfering pulses decrease gradually with the increase in the number of interfering pulses.

### 3.2. Validating the Effectiveness of the Secondary Sorting Algorithm

To verify the effectiveness of the second-level sorting algorithm, the pulse PDW of radar 7 in dataset 2 is first deleted; at this time, dataset 2 has 715 pulses. The IGWOA-DBSCAN algorithm is then used for binning, and the center of mass of each binned cluster in the binning result is calculated as the preprocessing of the second-level binning. Noise is added to each pulse of dataset 2, and then the pulse of radar 1 is added after the pulse of radar 8 as dataset 3. Dataset 3 has 1115 pulses. Set the pulse window size to 150 pulses. If a binned cluster has no pulse input for more than 150 pulses, the binned cluster is assigned to the inactive library.

**Figure 13:**
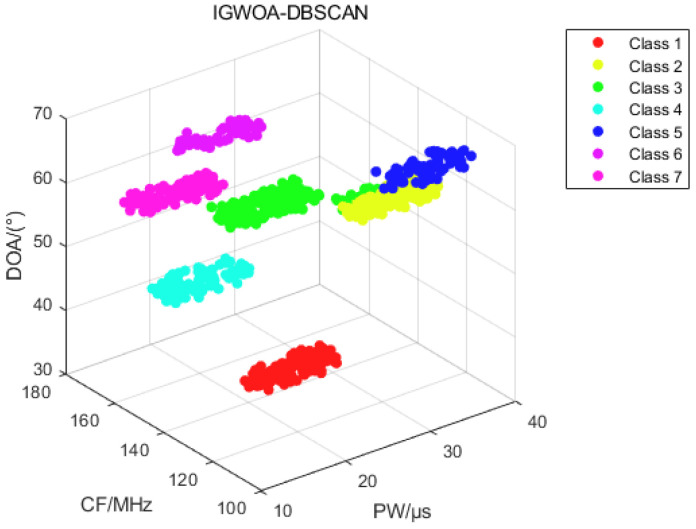
Distribution of radar pulses for primary sorting.

When sorting dataset 2, which contains only seven radars, the IGWOA optimization algorithm is used to find the optimal parameters for the DBSCAN algorithm proposed in Section 2.2. The number of iterations for the IGWOA optimization algorithm was set to 100, and the population size was set to 20. The parameters obtained through the IGWOA optimization algorithm were (8.025, 30, 3). After inputting these parameters into the DBSCAN optimization algorithm proposed in Section 2.2, Figure 13 was obtained.

**Figure 14:**
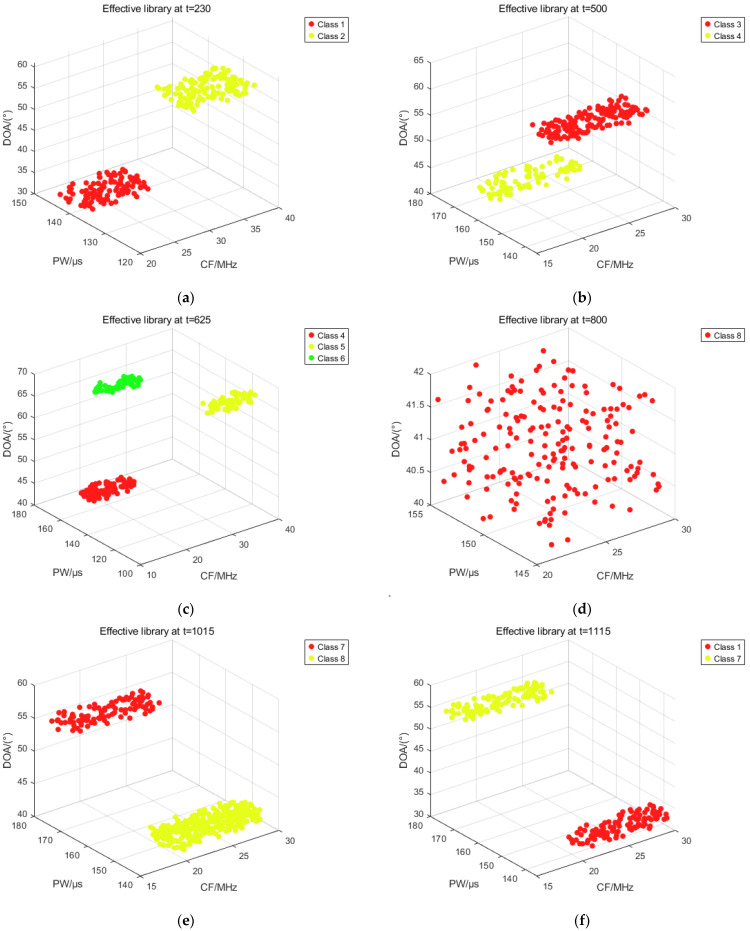
Distribution of effective library pulses for each representative moment. (**a**) Pulse distribution in the effective library at t = 230; (**b**) Pulse distribution in the effective library at t = 500; (**c**) Pulse distribution in the effective library at t = 625; (**d**) Pulse distribution in the effective library at t = 800; (**e**) Pulse distribution in the effective library at t = 1015; (**f**) Pulse distribution in the effective library at t = 1115.

In the pulse-by-pulse binning, the 230th, 500th, 625th, 800th, 1015th, and 1115th pulses are selected as representative moments, and the pulse distributions of the effective, inactive, and outlier banks are plotted for each representative moment. The simulation results are shown in Figure 14.

From the above figure, the distribution of effective library pulses at each representative moment can be obtained. At each representative moment, and to which sorting cluster the pulses in its pulse window are sorted, all the pulses of that sorting cluster are put into the effective bank. From this, it can be seen that at t = 230, the pulse window covers the 81st pulse to the 230th pulse. It is also known that the 81st to 100th pulses are the pulses of radar 1 due to the table of parameters of each radar pulse in dataset 2 in 3.1. Therefore, upon successful binning of the 81st to 100th pulses to Binning Cluster 1, all the pulses in Binning Cluster 1 appear in the effective library. In addition, the 101st pulse to 230th pulse undergoes sorting into sorting cluster 2, so all the pulses in sorting cluster 2 likewise appear in the effective library, and hence all the pulses possessing both sorting cluster 1 and sorting cluster 2 are present in the effective library at t = 230. The same is true for all the other subgraphs.

**Figure 15:**
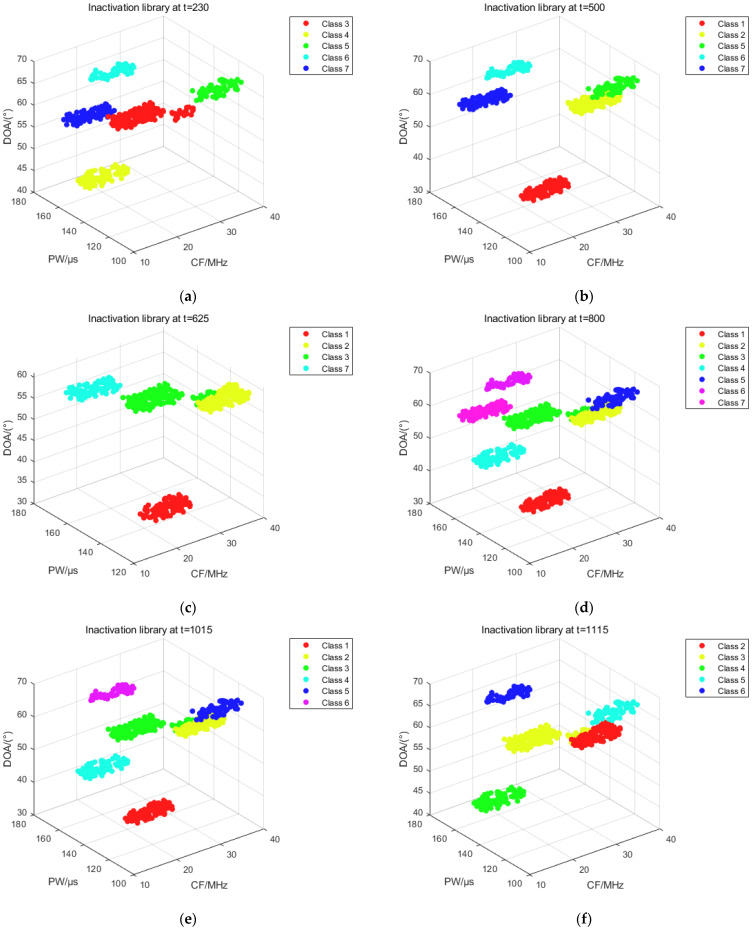
Distribution of inactivation library pulses for each representative moment. (**a**) Pulse distribution in the inactivation library at t = 230; (**b**) Pulse distribution in the inactivation library at t = 500; (**c**) Pulse distribution in the inactivation library at t = 625; (**d**) Pulse distribution in the inactivation library at t = 800; (**e**) Pulse distribution in the inactivation library at t = 1015; (**f**) Pulse distribution in the inactivation library at t = 1115.

The distribution of pulses in the inactivation library at each representative moment can be obtained from Figure 15. The pulse of each sorting cluster in the inactivation library consists of two parts. Taking subfigure b as an example, effective sorting cluster 1 and sorting cluster 2 have been inactive at t = 500, at which time sorting cluster 1 and sorting cluster 2 contain pulses input to the above sorting clusters at the first level of sorting, as well as pulses flowing in at the second level of sorting. Sorting clusters 5 through 7 contains only the pulses entered into these clusters during primary sorting. The same applies to the other subfigures.

**Figure 16:**
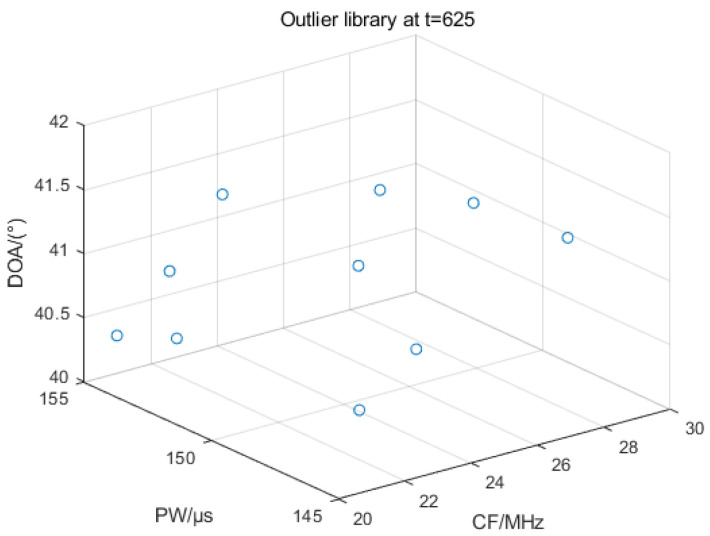
Outlier library pulse distribution at t = 625.

Figure 16 shows the distribution of outlier library pulses at t = 625. If the distance between the inflowing pulse and the center of mass of each sorted cluster in both the effective library and the outlier library is greater than the threshold value during online sorting, the pulse enters the outlier library. If the number of pulses is greater than the threshold MinPts, the IGWOA-DBSCAN algorithm is used to sort the pulses in the outlier library, and if the sorting is successful, new sorting clusters are added to the effective library, and pulses that have completed the sorting are deleted from the outlier library. The above figure shows the pulse distribution when the number of pulses in the outlier library is less than the threshold.

The statistics of the effective library, inactivation library, and outlier library in each representative moment are shown in Table 4.

## 4. Discussion

In this paper, based on the case of multiple radars transmitting the same waveform, the neighbor parameters are added based on the traditional DBSCAN algorithm, so that the spatial neighborhood of this algorithm can be adjusted more flexibly to improve the sorting ability. In the face of the preset parameters of the DBSCAN algorithm, the IGWOA algorithm is proposed to find the optimal preset parameters of this sorting algorithm. This algorithm effectively avoids the disadvantage of the traditional WOA algorithm falling into local optimization too early, increases the global search time, and obtains the global optimal solution as much as possible. The stream cluster sorting algorithm is used for online sorting after the pre-sorting is completed, and the concepts of effective library, inactivation library, and outlier library are introduced, which can effectively tap the dynamic change characteristics of pulse information. Simulation validation shows that the algorithm is able to effectively sort the data in the face of multiple radars transmitting the same waveforms and accurately mine the dynamic change characteristics of pulse information in the stream cluster sorting stage. This method improves the limitation that the traditional PRI sorting method has weak sorting ability in the face of complex radiation source signals, which is currently only applicable to conventional radiation sources.

Future research will focus on the collaborative sorting of PDW data from sensors located in different geographical locations and operating at different frequencies. The primary focus will be on improving the ability to identify and locate complex radiation source networks with enhanced accuracy.

## Figures and Tables

**Table 1:** Dataset 1 parameter settings for each radar.

Radar Catalogs	CF/MHz	PW/μs	DOA (°)	Pulse Number
Radar 1	97–103	41–43	5–7	200
Radar 2	147–153	36–38	9–11	200
Radar 3	177–183	41–43	4–6	200
Radar 4	197–203	34–37	8–10	200
Radar 5	297–303	36–38	15–17	200

**Table 2:** Dataset 2 parameter settings for each radar.

Radar Catalogs	CF/MHz	PW/μs	DOA (°)	Pulse Number
Radar 1	135–145	20–30	32–34	100
Radar 2	120–130	27–37	59–61	170
Radar 3	135–145	17–27	59–61	150
Radar 4	165–175	18–28	40–42	90
Radar 5	105–115	27–37	67–69	60
Radar 6	150–160	15–25	67–69	45
Radar 7	145–155	20–30	40–42	300
Radar 8	165–175	15–25	55–57	100

**Table 3:** Accuracy of sorting by each algorithm in dataset 2.

Performance Evaluation	DBSCAN	WOA-DBSCAN	IGWOA-DBSCAN
**Accuracy rate**	40.1%	60%	97.8%

**Table 4:** Number of pulses in each bank at each representative moment.

Representative Moments	Number of Effective Library Pulses	Number of Pulses in the Inactivation Library	Number of Pulses in the Outlier Library
230	230	468	0
500	230	722	0
625	195	940	10
800	185	1330	0
1015	400	1230	0
1115	300	1330	0

## Data Availability

Dataset 1 is available in the Simulation section of Chinese Reference [28]. Dataset 2 is obtained from dataset morphing in Reference [29]. The download link for Reference [29] has been placed in the Supplementary Materials section.

## Supplementary Materials

The following supporting information can be downloaded at: https://www.scidb.cn/en/detail?dataSetId=22dee7d7658e4c95b9a57d3b9666e0d7 (accessed on 18 August 2025), Dataset 2: PDW Datasets.

## Abbreviations

The following abbreviations are used in this manuscript:

DBSCAN	Density-based spatial clustering of applications with noise
WOA	Whale optimization algorithm
IGWOA	Improved golden sine whale optimization algorithm
